# A Multiparent Advanced Generation Inter-Cross to Fine-Map Quantitative Traits in *Arabidopsis thaliana*

**DOI:** 10.1371/journal.pgen.1000551

**Published:** 2009-07-10

**Authors:** Paula X. Kover, William Valdar, Joseph Trakalo, Nora Scarcelli, Ian M. Ehrenreich, Michael D. Purugganan, Caroline Durrant, Richard Mott

**Affiliations:** 1 Department of Biology and Biochemistry, University of Bath, Bath, United Kingdom; 2 Faculty of Life Sciences, University of Manchester, Manchester, United Kingdom; 3 Wellcome Trust Centre for Human Genetics, University of Oxford, Oxford, United Kingdom; 4 Center for Genomics and Systems Biology, New York University, New York, New York, United States of America; University of Georgia, United States of America

## Abstract

Identifying natural allelic variation that underlies quantitative trait variation remains a fundamental problem in genetics. Most studies have employed either simple synthetic populations with restricted allelic variation or performed association mapping on a sample of naturally occurring haplotypes. Both of these approaches have some limitations, therefore alternative resources for the genetic dissection of complex traits continue to be sought. Here we describe one such alternative, the Multiparent Advanced Generation Inter-Cross (MAGIC). This approach is expected to improve the precision with which QTL can be mapped, improving the outlook for QTL cloning. Here, we present the first panel of MAGIC lines developed: a set of 527 recombinant inbred lines (RILs) descended from a heterogeneous stock of 19 intermated accessions of the plant *Arabidopsis thaliana*. These lines and the 19 founders were genotyped with 1,260 single nucleotide polymorphisms and phenotyped for development-related traits. Analytical methods were developed to fine-map quantitative trait loci (QTL) in the MAGIC lines by reconstructing the genome of each line as a mosaic of the founders. We show by simulation that QTL explaining 10% of the phenotypic variance will be detected in most situations with an average mapping error of about 300 kb, and that if the number of lines were doubled the mapping error would be under 200 kb. We also show how the power to detect a QTL and the mapping accuracy vary, depending on QTL location. We demonstrate the utility of this new mapping population by mapping several known QTL with high precision and by finding novel QTL for germination data and bolting time. Our results provide strong support for similar ongoing efforts to produce MAGIC lines in other organisms.

## Author Summary

Most traits of economic and evolutionary interest vary quantitatively and have multiple genes affecting their expression. Dissecting the genetic basis of such traits is crucial for the improvement of crops and management of diseases. Here, we develop a new resource to identify genes underlying such quantitative traits in *Arabidopsis thaliana*, a genetic model organism in plants. We show that using a large population of inbred lines derived from intercrossing 19 parents, we can localize the genes underlying quantitative traits better than with existing methods. Using these lines, we were able to replicate the identification of previously known genes that affect developmental traits in *A. thaliana* and identify some new ones. This paper also presents all the necessary biological and computational material necessary for the scientific community to use these lines in their own research. Our results suggest that the use of lines derived from a multiparent advanced generation inter-cross (MAGIC lines) should be very useful in other organisms.

## Introduction

Most plant traits of agronomic and economic interest, such as seed dormancy, flowering time, fruit production, disease resistance, etc., vary quantitatively and have complex genetic inheritance. Their phenotypic expression is determined by the combination of many genetic and environmental factors. Naturally occurring genetic variation is a valuable source of alleles for economically important traits, but much of the genetic basis of natural variation in these traits remains unresolved [1],[2]. Thus, new resources to dissect and exploit this variation are needed.

*Arabidopsis thaliana* is an ideal species in which to develop resources because it is a model for the study of plant genetics, and extensive natural variation segregates among accessions of *A. thaliana* for many ecological and developmental traits [3]–[5]. In addition, an extensive repertoire of genomic tools facilitate the cloning of quantitative trait loci (QTL) [6]–[8]. Because *A. thaliana* is in the same family as a number of important crops (rape seed, cabbage, broccoli and other brassicas), identification of causal genes may lead to the identification of homologous loci important for improving crop quality and productivity [9]–[11], as well as have broader applications [12].

The populations of *A. thaliana* used for genetic mapping so far can be classified into naturally occurring inbred lines (accessions) and synthetic populations. Genetic association in the former is a more recent development [13], whilst the mapping of QTL in the latter is well established [14]–[16]. Synthetic populations include F2, backcrosses, recombinant inbred lines (RILs) and advanced intercross lines (AIL), all created from a cross between two accessions that differ for the trait of interest (reviewed in [17], and [18]); many QTL for complex traits have been mapped using these crosses and RILs. Their two advantages are that the power to detect a QTL segregating in a two-allele system is high, and that synthetic populations usually have no population substructure. The power to detect a QTL in any mapping population depends on the fraction of the phenotypic variance it explains. If the QTL is diallelic then this is proportional to *p*(1−*p*), where *p* is the minor allele frequency at the QTL. This quantity is greatest when *p* = 0.5, as is approximately the case in the majority of synthetic populations descended from two parental lines. The lack of substructure means there are few long-range correlations between genotypes and consequently the QTL can be mapped independently, with little risk of false positive “ghost” QTL. The main disadvantage is poor mapping resolution: QTL identified using these designs typically have confidence intervals of 5 to 20 cM [19]–[21], corresponding on average to 1.2 to 4.8 Mb and covering hundreds of candidate genes.

Genetic association using naturally occurring accessions has complementary strengths and weaknesses: minor allele frequencies underlying a QTL are rarely close to 0.5, with many rare alleles [22], so the QTL discovery rate is not as efficient. However, the advantage of association mapping is its higher mapping resolution; because linkage disequilibrium decays very quickly in natural accessions, it is sometimes feasible to map QTL to near single-gene resolution [23]. The main challenge for association studies at the moment is population sub-structure (due to demographic causes), which requires more sophisticated analyses such as linear mixed models [13],[24] to control for false positives.

In classical synthetic populations further fine-mapping is required before QTL can be cloned, which is slow and expensive. In addition, only a limited number of QTL may be identified within each cross, since only QTL for which the two accessions differ can be detected. The limited scope of each QTL study means that mapping the same trait in different panels of RILs commonly yields different QTL [21], [25]–[27], and it is not possible to investigate interactions between QTL identified in different panels. More than two alleles are likely to segregate per locus, and the direction of QTL effects may vary depending on the genetic background due to epistasis and pleiotropy [20] and gene by environment interactions [28]. Therefore simple synthetic populations do not capture the full genetic architecture of complex traits.

The use of heterogeneous stocks (HS) improves the power to detect and localise QTL, and model genetic architecture more realistically. HS are the result of repeated crosses between multiple parental lines over many generations to produce a highly recombinant heterozygous outbred population. This strategy has been successfully used for fine-mapping QTL using eight parental strains in mice [29],[30] and Drosophila [31]. A disadvantage with HS is that each individual's genome is unique and heterozygous, and therefore the population must be genotyped at high density each time it is phenotyped. A related strategy, that avoids the need to re-genotype, is to generate RILs from multiple parents [32],[33], where the genomes of the founders are first mixed by several rounds of mating and then inbred to generate a stable panel of inbred lines. The name MAGIC (for multiparent advanced generation intercross) has been suggested for this type of population [33]. The large number of parental accessions increases the allelic and phenotypic diversity over traditional RILs, potentially increasing the number of QTL that segregate in the cross. The larger number of accumulated recombination events increase the mapping accuracy of the detected QTL compared to an F2 cross [34]. Thus, MAGIC lines occupy an intermediate niche between naturally occurring accessions and existing synthetic populations.

Here we present the first set of MAGIC lines. They are derived from an advanced intercross of *Arabidopsis thaliana* produced by intermating 19 natural accessions for four generations (as described in [35]) and then inbreeding for 6 generations. The resulting nearly homozygous lines form a stable panel of RIL that do not require repeated genotyping in each QTL study. We describe their construction and genomic structure, and demonstrate that these lines can be used for QTL fine-mapping using examples of developmental traits. Finally, we establish the statistical and computational tools and resources required for their analysis, and propose new candidate genes for germination date and bolting time.

## Results

### Construction of the MAGIC lines

The MAGIC lines were initiated by intermating the 19 “founder” accessions of *A. thaliana* listed in Table 1 for 4 generations as described in [35]. To avoid assortative mating during the mixing of the accessions, we used a staggered planting scheme and replanted families as needed to perform the randomly assigned crosses. The founders were selected either because they originate over a wide geographical distribution or are commonly used (i.e. Col-0 and Ler-0). The intermating produced 342 F4 outcrossed families. From each F4 family we derived up to 3 inbred MAGIC lines (MLs) by selfing an F4 plant for six generations. Lines derived from the same F4 can be thought of as “cousins”, as they are expected to share 25% of their genomes by descent.

**Table 1:** List of accessions used to found the MLs.

AIMS stock center #	Accession	Origin
CS6643	Bur-0	Ireland
CS6660	Can-0	Canary Isles
CS6673	Col-0	USA
CS6674	Ct-1	Italy
CS6688	Edi-0	Scotland
CS6736	Hi-0	Netherlands
CS6762	Kn-0	Lithuania
CS20	Ler-0	Germany
CS1380	Mt-0	Libya
CS6805	No-0	Germany
CS6824	Oy-0	Norway
CS6839	Po-0	Germany
CS6850	Rsch-4	Russia
CS6857	Sf-2	Spain
CS6874	Tsu-0	Japan
CS6889	Wil-2	Russia
CS6891	Ws-0	Russia
CS6897	Wu-0	Germany
CS6902	Zu-0	Germany

**Figure 1:**
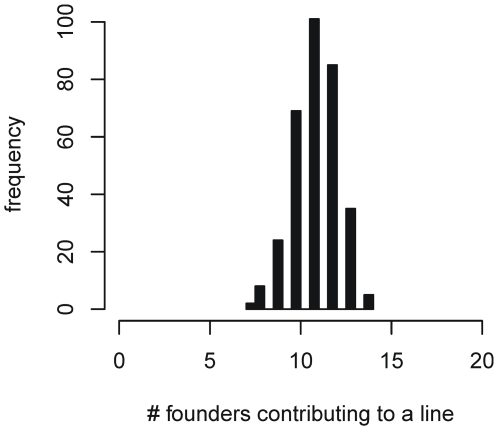
The distribution of the number of distinct founder genomes contributing to a ML. Each ML is descended through a funnel mating design from up to 16 distinct founder genomes. This histogram shows the fraction of lines descended from a given number of distinct founders.

Given the random mating design, each F4 family incorporates a variable number of accessions in their pedigree, with an average of 9.97 distinct founder accessions per F4 (the distribution is plotted in Figure 1); Table S1 lists the lines, the cross they were derived from and which accessions contribute to their pedigree. Although there are 1026 MLs in production, in this paper we focus on a subset of up to 527 lines for which genotype data is currently available (the exact number of lines phenotyped varies for each trait). The ML germplasm is being made available through the Arabidopsis stock centre (http://www.arabidopsis.org).

### Phenotypic variation among MAGIC lines

Each ML was planted in 5 replicate pots, and grown in a greenhouse or growth chambers. The frequencies of lines expressing the qualitative trait “glabrous” (i.e. whether their leaves were completely devoid of trichomes, which would have been derived from accession Wil-2) or “erecta” (i.e. had a compact inflorescence with sword shaped fruits, typical of accession Ler-0) were 4.4% and 7.2% respectively, close to the expected frequency of 1/19 (5.2%).

Extensive variation was observed for developmental quantitative traits (see Table 2). For these traits, we measured the heritability 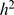 among MLs in two ways: (i) 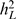 is the proportion of variation that is due to genetic differences between lines, using the phenotypic average of the replicates within each line. (ii) 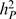 is an estimate of the genetic variance if only one replicate per line were phenotyped. Thus 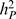 measures the true genetic variance between individual plants, while 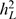 is the effective genetic variance in an experiment with replication (as is the case in this study). In all cases 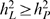 and 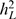 increases with the number of replicates. These estimates are given in Table 2 along with the sample sizes for each trait. As highly inbred lines were used, within-line variability is almost entirely non-genetic, and hence the mean of each line was used for QTL mapping. Therefore 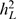 is the upper bound of 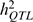, the fraction of the variance that is due to mapped QTL; 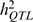 indicates how much genetic variability has been found by mapping.

**Table 2:** Range in measured phenotypes and heritabilities for the traits measured.

Trait	Range	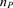	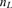	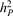	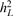	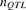	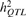
Days to germination	4–31	2227	433	0.50	0.84	1.94	27.34
Growth rate	−9–17^b^	1706	351	0.22	0.46	2.05	28.02
Days to bolt	13–85	2202	433	0.72	0.93	3.63	63.70
Days from bolt to flower	3–37	2176	433	0.40	0.76	2.32	33.26
Days to Flower (LD)	21–126	1228	336	0.58	0.81	3.04	55.12
Days to Flower (SD)	33–128	1104	323	0.54	0.78	3.69	63.60
RLN (Long Day)	8–96	1228	336	0.58	0.81	3.27	60.26
RLN (ShortDay)	8–181	1104	323	0.25	0.51	3.58	56.19
erecta		2412	465	0.73		1.00	80.36^a^
glabrous		2412	465	0.77		1.00	87.09^a^

a For binary traits 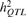 is estimated as the fraction of the deviance.

b Growth rate is the residual of the number of leaves at day 28 after regression on the number days to germination; hence the minimum value in the range is negative.

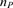
 is the number of plants phenotyped for the trait, 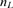 is the number of MLs. 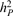 is the estimated heritability between plants and 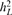 the estimated heritability between lines. 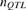 is the average number of QTL found in multiple QTL models fitted to 500 resampled data sets. 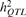 is the average fraction of variance accounted for by the multiple QTL models. LD = Long day; SD = short days and RLN (Rosette leaf number).

### Genetic variation among the 19 founder accessions

**Figure 2:**
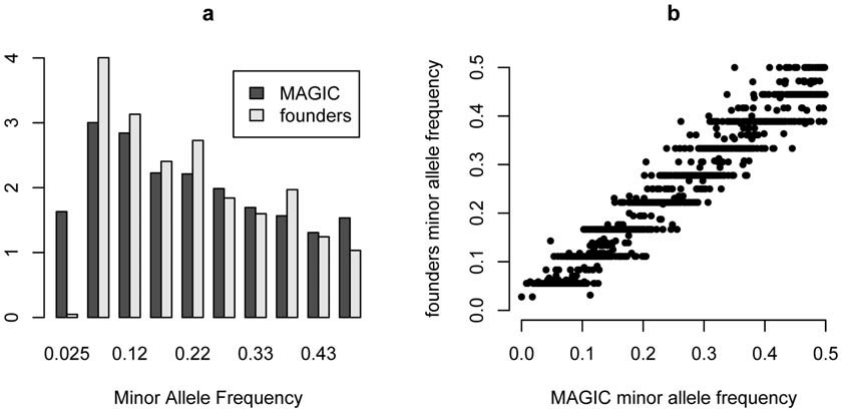
Distribution of the minor allele frequency for the 1,260 genotyped SNPs. (A) in the 19 founders; (B) in 527 MLs.

**Figure 3:**
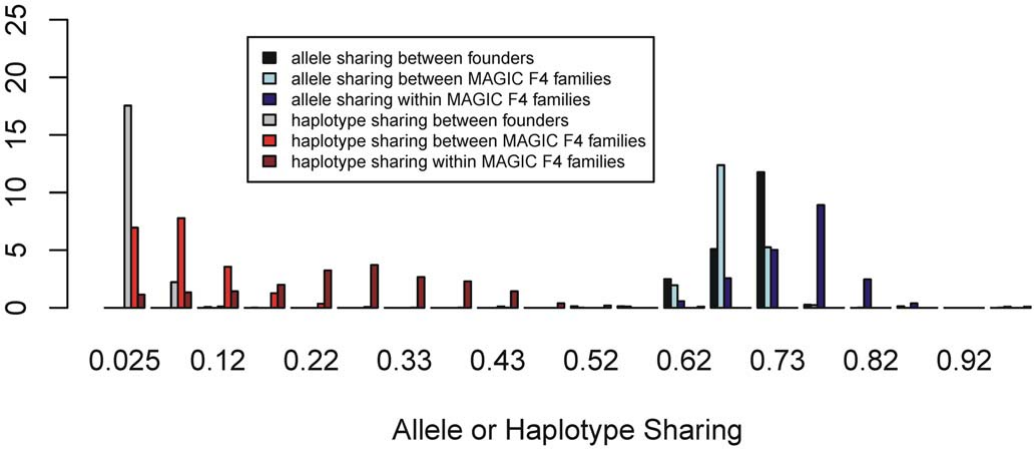
Distribution of allele and 10-SNP Haplotype sharing among the 19 founders and the MLs. Sharing between and within F4 families are plotted separately.

For the 1260 SNPs for which all MLs were genotyped, the minor allele frequency was 0.22 in the founders; the distribution of allele frequencies is shown in Figure 2. On average 70% of SNPs are shared between any pair of founders, and each founder is about equidistant from the others (Figure 3). The two exceptions are Col-0 and Ler-0, which share only 52% SNPs, most likely due to bias in SNP ascertainment (see Materials and Methods); and the closely related pair Oy-0 and Po-0, for which 86% of alleles are shared. Po-0 has higher heterozyogosity (5.4%) than for any of the other founders (range 0% to 0.7%), suggesting it is a hybrid. This finding did not result from DNA contamination, as it was replicated when Po-0 was re-genotyped separately from other accessions. We also genotyped DNA from the original seed stocks received from the Arabidopsis stock center, and ruled out germplasm contamination during the study.

**Figure 4:**
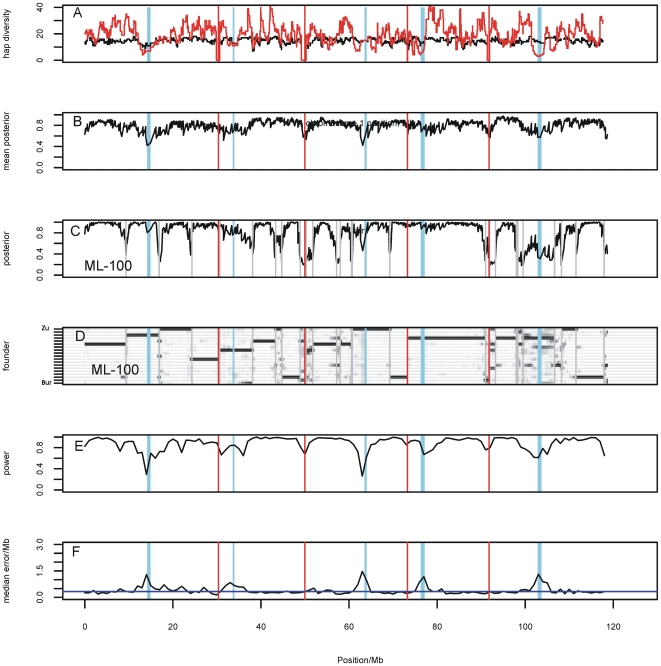
Genome-wide properties of the MLs. In each panel the x-axis represents the complete 120 Mb genome of *A. thaliana*, with vertical red lines marking the chromosome boundaries and the pale blue vertical bars indicating the centromeres. (A) Number of 10-SNP haplotypes observed among founders (black) and MLs (red) across the genome. (B) The maximum posterior founder probability, *m_Li_* at locus *L*, averaged across all MLs *i*. (C) The maximum posterior founder probability, *m_Li_* for the ML *i* = “ML-100”. The vertical grey lines indicate probable recombination breakpoints where the identity of the most probable founder changes. (D) The posterior founder probabilities for ML-100. The vertical axis represents the 19 possible founders, s, in alphabetical order. The probability 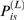 for founder *s* at locus *L* is represented by a grey bar at coordinate (*L,s*), the shade of grey varying from white (*P* = 0) to black (*P* = 1). (E) The locus-specific power to detect a QTL explaining 10% of the phenotypic variance, from 40,000 simulations. In each simulation a 10% QTL was placed randomly along the genome. Successful detection is defined as the event that the genome-wide maximum in the genome scan for the QTL is within 3 Mb of the true QTL location. (F) The locus-specific median mapping error for the successfully detected QTL in (E).

We measured the local haplotypic diversity among the founders using a moving window of k adjacent SNPs. With k = 5 (corresponding to a genomic interval of approximately 400 kb), the founders partition into 8.5 distinct haplotypes on average, and into 14.3 with k = 10. The number of distinct haplotypes using k = 10 across the genome is plotted in Figure 4A. The variation appears sporadic without large-scale structure, except for an apparent loss of variability around the centromeres.

### Genetic variation among the MAGIC lines

The average SNP minor allele frequency in the MLs is also 0.22, and is distributed similarly to that in the founders (Figure 3), with the exception that there are fewer alleles with intermediate frequencies, as expected by drift. The extent of allele sharing between MLs was consistent with their breeding history. Cousin MLs, descended from the same F4 family, share 74% of alleles on average whilst those from different F4 families share 68% (Figure 3). Thus, as expected, cousins share slightly more alleles than the founders and non-cousins slightly less. We found 19 pairs of lines that share over 95% of their genotypes, and three pairs were identical. We believe this is most likely due to errors during breeding; these 38 lines were therefore omitted from the heritability analysis and QTL mapping.

We found that SNP-sharing is only a weak predictor of haplotype sharing. The distribution of 10-SNP haplotype-sharing percentage between the MLs is also plotted in Figure 3. MLs descended from different F4 families share on average 7.5% of 10-SNP haplotypes. This suggests that the genotyped SNPs separate the 19 founders into about 14 10-SNP haplotypes (1/14 = 7.1%). On average haplotype sharing among cousin lines is 25.4%, which is very close to the expected degree of 25% identity by descent. Haplotype-sharing between founders is very small, with mean 2.5%, as would be expected since linkage disequilibrium among accessions of *A. thaliana* breaks down, on average, within 10 kb [36]. The spatial distribution of 10-SNP haplotype diversity in the MLs does not track that in the founders except in regions (such as the centromeres) where there is a reduction in haplotype diversity. In general there are more ML haplotypes present at a locus because recombination breaks up the founder haplotypes (Figure 4A).

**Figure 5:**
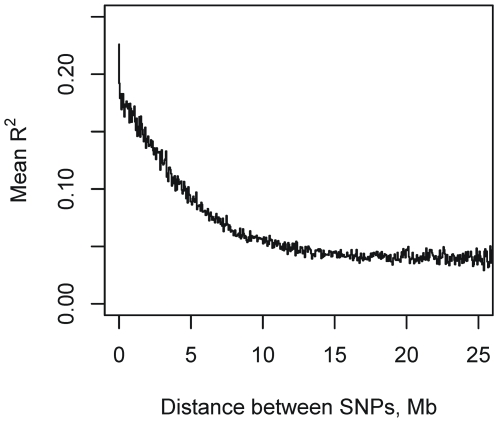
Distribution of the decay in mean LD (R^2^) as a function of distance between SNPs in the MLs.

**Figure 6:**
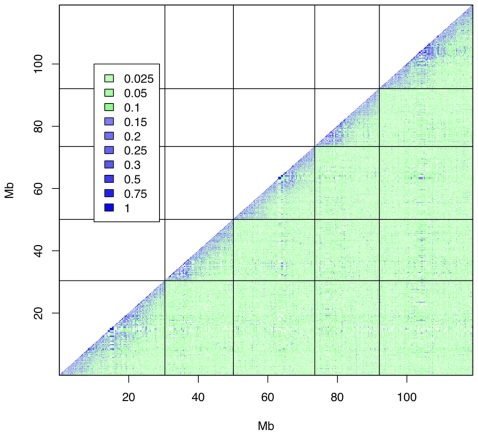
Genome-wide patterns of LD (R^2^) in the MLs. The chromosome boundaries are marked by black lines. The intensity of the LD between SNPs at loci x,y is indicated by the colour in the corresponding x,y coordinate, using the scale indicated in the legend.

The average decay in linkage disequilibrium (LD) in the MLs, as measured by the correlation R2, is plotted as a function of distance in Figure 5. The mean correlation between SNPs decays to 0.17 by about 0.5 Mb, and approaches the background level of ∼0.05 by about 15 Mb. The genome-wide distribution of R^2^ is plotted in Figure 6 and shows that there is minimal LD between chromosomes. For SNPs on different chromosomes, the mean value of R^2^ is 0.04; it exceeds 0.5 for 0.00016% of SNP pairs and exceeds 0.15 for 0.5% of pairs. These results suggest first that the QTL mapping resolution should be under 500 kb, and second that population structure in the MLs is unlikely to give rise to ghost QTL due to genotype correlations between chromosomes (see simulations). Consistent with other studies, we found that among the founders, mean R^2^ decays within 10 kb (data not shown).

The six generations of selfing used to generate each ML should produce genomes that are nearly homozygous. We identified 32 regions of residual heterozygosity in 29 MLs, defined as loci spanning at least 10 SNPs (ranging in length from 287 kb to 2.8 Mb) in which the density of heterozygotes exceeded 50%. Six of these were more extensive regions spanning at least 20 SNPs, the largest spanning 36 SNPs. Thus, at the level of resolution visible by the current genotype density, only about 1% of 527 ML exhibit residual heterozygosity, extending over about 1% of their genomes. Therefore, for the purposes of QTL mapping, we neglected all heterozygous genotypes.

### QTL Mapping

There are many statistical methods for mapping QTL in diallelic populations such as F2 crosses, advanced intercrosses and RILs descended from two parents. These methods are optimized to exploit the simplicity of the diallelic genetics. However, the analysis of multi-parental populations requires a different approach, because single marker association or interval mapping can fail to detect a QTL if the causative alleles do not segregate between the founders in the same way as the individual markers [37]. Furthermore, when mapping QTL in structured populations, as is the case here, the evidence for the existence of a QTL has to be considered in the context of other QTL which might explain some of the same component of variation [30]. Population structure can produce long-range correlations between genotypes and hence “ghost” QTL, although the LD analysis suggests that the MAGIC population is relatively immune to this phenomenon.

To deal with these issues, we apply three QTL mapping methods. The first two approaches use fixed-effects QTL models but accommodate population structure, in different ways, either by multiple-QTL modeling or by including random effects to explain correlations introduced by population structure. The third addresses the problem of the large number of parameters required in a fixed effects model, by introducing a hierarchical Bayesian random effects model. All approaches model the mosaic structure of the MAGIC genomes as described in [30],[37],[38] and implemented in the R package HAPPY. We also investigated the simple alternative of single-marker association, but the results are not presented in detail here. The genome scans from all methods can be viewed through our genome scan browser, at http://gscan.well.ox.ac.uk/arabidopsis/wwwqtl.cgi.

In complex trait analysis, multiple QTL of small individual effect are expected to segregate, and the evidence supporting a given QTL will depend on which other QTL are included at the same time. The variation explained by different QTL can overlap, especially when there is significant population structure, and which can generate false positive “ghost” QTL. Therefore, evidence for each QTL is evaluated in the context of many different multiple QTL models in the three step process described in more detail in Materials and Methods.

In the first step, a probabilistic reconstruction of the haplotype mosaic of each ML was calculated, taking into account information from multiple markers and the genetic map. Using a hidden Markov model, we computed the probability 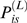 that the founder is *s* at the locus *L* for individual *i*. The maximum posterior 
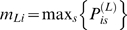
 measures the certainty of the reconstruction at a locus *L* for individual *i*; high certainty, when 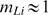, implies most of the probability is concentrated on a single founder. The mean of 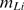 across all SNPs and MLs is 0.83, and 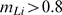 at 72% of loci (Figure 5B). Ambiguities generally occur near the chromosome boundaries and the centromeres. Figure 5C and 5D shows the probabilistic reconstruction of a typical line, ML-100, and shows that except near recombination breakpoints the identity of the founder haplotype is usually known with high probability. These results suggest that increasing the density of SNPs would not significantly improve the haplotype reconstruction, except possibly near the centromeres, where it is unclear if the loss of haplotypic diversity in Figure 5A is genuine or is due to SNP ascertainment. The relatively high density of SNPs used here is already 5–10 times greater than for other RILs.

In a second step, the genome is scanned for evidence for a QTL in each SNP interval using a fixed effects model, and ignoring the effects of other QTL. This corresponds to a standard genome scan. Simulations were used to estimate genome-wide thresholds for statistical significance when no QTL were present. We found that on average the genome-wide maximum logP_MAX_ = 2.8, and 95% of scans satisfy logP_MAX_<3.52. Thus linkage disequilibrium causes the 1255 marker intervals that are tested to behave like about 10^2.8^ = 630 independent tests (as the expected most extreme p-value from N independent tests is 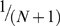. We used logP = 3 as a threshold in the multiple QTL modeling described below.

Simulations also show that both the power to detect a QTL and the expected mapping resolution are weakest near the centromeres and chromosome ends, but are fairly uniform across the rest of the genome (Figure 5E and 5F). Both quantities also depend on the effect size of the QTL. For example, the power to detect a QTL accounting for 10% of between-line phenotypic variance is close to 1 except at the centromeres (overall median 0.93) and the median mapping resolution (defined as the distance between the locations of the true and predicted QTL) is 0.33 Mb, whereas for a 5% QTL the median power is 0.52 and resolution 0.56 Mb. Thus, as a guide to future QTL-mapping studies using the same number of ∼460 lines phenotyped here, the transition zone for reliable detection and fine-mapping lies between QTL effect sizes of 5% and 10%. Note that these are effect sizes for the mean phenotypic value over the replicates within each line, not the effect size in individual plants, so increasing the level of replication would improve the power to detect and fine-map QTL of small effect.

We also investigated the power and accuracy that would be achievable if the complete MAGIC population of 1026 lines were used, by simulating an instance of the full cohort. We found the power to detect a QTL that explains 5% of the variation increased to 79% and the median mapping resolution was reduced to 0.29 Mb. The corresponding figures for a QTL that explains 10% of the variation were 96% and 0.19 Mb.

**Figure 7:**
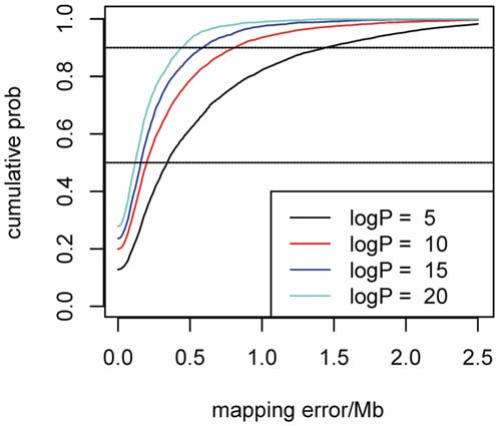
Distributions of the mapping error in QTL location for QTL in which logP_MAX_ is 5, 10, 15, or 20. Each curve is estimated from simulations as described in Materials and Methods. The width of the corresponding confidence interval is twice the mapping error. The horizontal dashed lines cut the distributions at the 50% (lower) and 90% (upper) points.

Because QTL effect size is not a direct measure of statistical significance (i.e., the logP corresponding to a given effect size varies), the distribution of the width of QTL confidence intervals was modeled instead as a function of the peak height logP_MAX_ at the locus (see Materials and Methods). Figure 7 shows the distributions for the mapping error (i.e., half the width of the confidence interval) for a range of logP_MAX_ values, and Table 3 gives 90% confidence intervals for the QTL mapped in this study.

**Table 3:** List of QTL identified and their location.

Phenotype	SNP	Position	logP	EQ	chr	90% CI	
						lower	Upper
Days to germ	MN3_15977654	15987185	6.46	1	3	14847114	17108129
	MN4_1553589	1569395	3.04	0.944	4	0	3406581
Growth rate	MASC00349	12006105	3.12	0.85	2	9976948	13672649
	MASC04651	903706	3.11	1.192	4	0	2751043
Days to bolt	MASC00497	20335845	5.03	0.26	1	18885610	21742617
	NMSNP1-24738247	24745092	4.36	0.89	1	23166542	26309953
	FRI_2343	286096	20.07	1	4	0	709276
	MN5_3491425	3494784	9.18	1.02	5	2646180	4336671
Days to Flower (LD)	MN1_21908389	21926683	3.23	0.582	1	20086750	23730457
	MN5_476404	476595	7.32	2.272	5	0	1493246
Days to Flower (SD)	MASC00545	19174205	3.08	0.254	1	17097612	20797655
	MN4_428535	428637	3.56	0.98	4	0	2198574
	NMSNP4-10977574	10989679	5.09	1.02	4	9558787	12396362
	MASC03480	890796	6.86	0.272	5	0	1820618
	MN5_4318001	4318184	9.35	0.834	5	3481276	5154727
RLN (LD)	MN4_241821	255541	4.13	1.062	4	0	1864192
	MN5_1399718	1399835	11.09	1.966	5	681914	2117515
RLN (SD)	PHYE_1561	10094177	3.05	0.588	4	8189089	11901220
	MN5_3227635	3254762	4.80	1.306	5	1732469	4722802
	MN5_6222071	6222177	4.13	0.474	5	4599779	7844364
	MN5_22414731	22414852	4.23	0.784	5	20819417	24009986
Days from bolt to flower	MN4_142943	192383	6.32	1.002	4	0	1317117
	MN5_3289426	3309828	6.12	1.306	5	2085426	4493427
Erecta	MN2_11300378	11315919	27.45	0.998	2	10957708	11643037
Glabrous	MN3_10363610	10372891	24.01	0.998	3	9944539	10782684

Each row refers to one QTL. SNP is the identity of the left-hand SNP in the marker interval at the QTL peak; logP is −log_10_(ANOVA P-value) at the QTL peak; EQ is the expected number of QTL estimated from 500 resamples; chr is the chromosome and the 90% confidence interval (CI) for the QTL based on simulations are given in the final two columns.

We also investigated whether QTL are likely to generate “ghosts” on other chromosomes [39], from simulations with a single large-effect QTL explaining 15% of the variance. The distribution of the maximum logP on chromosomes other than that containing the QTL was very close to that of the null model with no QTL, (data not shown) indicating that inter-chromosomal LD is unlikely to generate false positives, and that the effects of MAGIC population structure on QTL are small.

Finally, the evidence in favour of a QTL is re-evaluated by resampling the data 500 times and fitting multiple QTL models. Each resampling produces a different set of QTL, and the fraction of models containing a given QTL is the measure of support for the QTL. Because the location of a QTL (defined as the marker interval with maximum logP in the region) may shift between resamples, we integrate the fraction over neighbouring loci, to estimate the expected number of QTL in the region, or EQ. Where this number is greater than 1, it suggests that more than one linked QTL is present. Table 2 lists the mean number of QTL identified for each trait, and the mean fraction of phenotypic variance explained by all QTL found for the trait, averaged across all sampled multiple-QTL models. It is unlikely all QTL are detected so this fraction should be less than 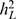. Table 3 lists the individual QTL with EQ>0.25, which was used in an earlier QTL study in mice [30] where it was shown by simulation to be a reasonable threshold. However, the interpretation of EQ as an indicator of a QTL depends, in a complex way, on the number and effect sizes of the other QTL present and on the population structure. Therefore it is difficult to give a simple interpretation of EQ as a probability of a QTL.

QTL heritability is defined as the fraction of variance accounted for by QTL, averaged across all sampled multiple-QTL models, and is given in Table 2. Overall, Table 2 indicates that, depending on the phenotype, up to 63% of the between-line heritability is accounted for by the mapped QTL. The sources of the missing heritability might include environmental interactions, undetected QTL with small genetic effects, and epistasis. Dominance effects should be negligible given the lines are effectively inbred.

### Linear Mixed Effects Model (Empirical Bayes) and Hierarchical Bayes QTL mapping

We also mapped QTL using two alternative methods. The first, an Empirical Bayes linear mixed effects model, assesses the evidence for a QTL taking into account the expected population structure. This class of method has been shown to be effective at controlling for population structure in association mapping with natural accessions of *A. thaliana* and in other species [13],[24]. Our model assumed that mean trait values on lines descended from the same F4 are likely to be more similar than otherwise. We found that the QTL logP values produced by this method were slightly smaller than the fixed effects model described above, but that the difference was generally negligible. This result, suggests again that population structure does not have a strong impact.

The second, Hierarchical Bayesian, method ignores population structure but models the founders' trait values at a QTL as random effects sampled from a Normal distribution. The variance of this distribution is expressed as 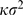 where 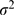 is the total phenotypic variance and 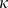 is the proportion of variance explained by the QTL. The rationale behind this approach was that the power to detect a QTL might be increased if only a single parameter 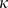 needed to be estimated, compared to up to 18 with a fixed effects model. We investigated several measures of the posterior evidence for a QTL, such as the log Bayes factor, and found the most useful was the posterior mode of 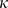, which tracks the variance explained by the QTL. The genome-wide threshold for 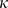 was calculated via simulation as 5.8%, which is close to the approximate minimum QTL variance (5%) at which the fixed effects model can detect QTL with 50% power. We found little difference between the Hierarchical Bayesian and the fixed effects analysis. Consequently, the remainder of the paper focuses on the fixed effects resampling methodology. However, the genome scans for all three methods are available from the GSCANDB browser http://gscan.well.ox.ac.uk/arabidopsis/wwwqtl.cgi. The browser also shows the results of standard single marker association (SMA). We do not report the results of SMA except to remark that in general they are harder to interpret than the haplotype-based tests because the significance of each tested SNP at a QTL can oscillate wildly depending on whether the allelic distribution pattern among the founder accessions matches that of the causative polymorphism.

### Successfully identified known genes associated with binary phenotypes

**Figure 8:**
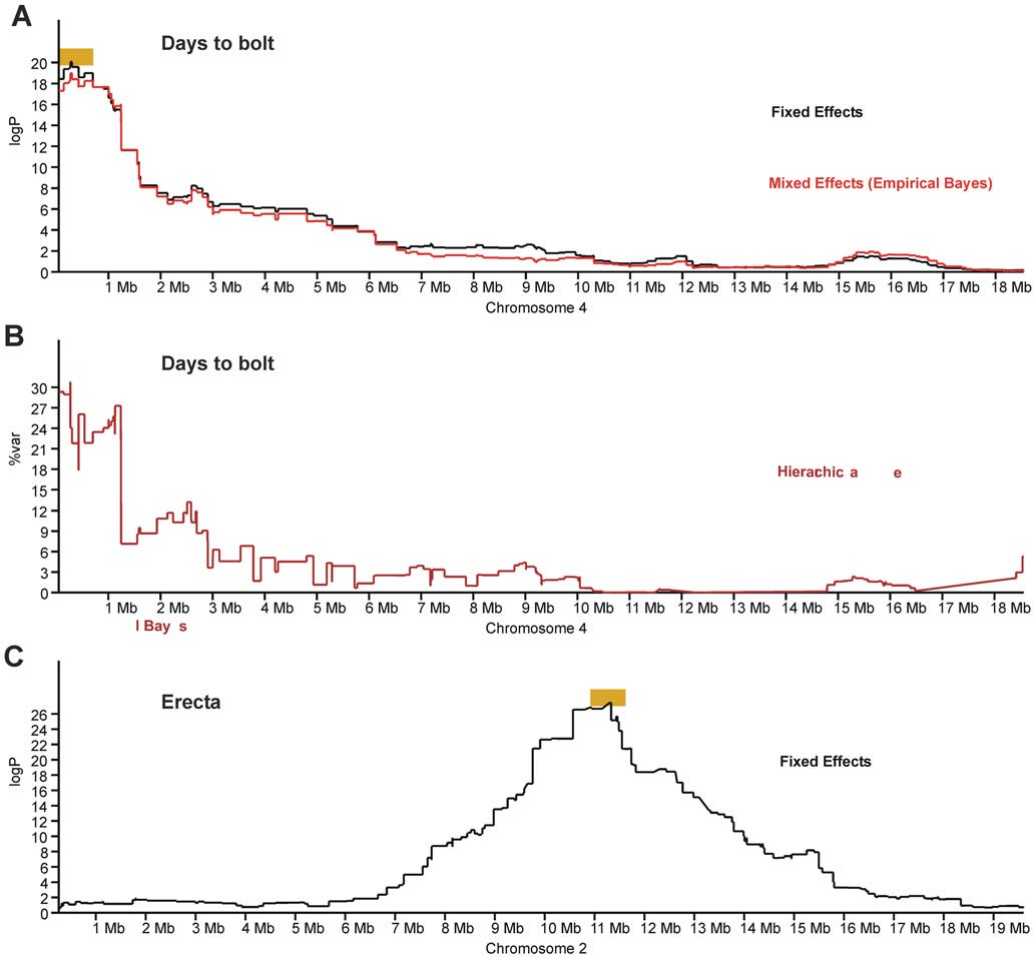
Examples of QTL scans. The orange bars indicate the 90% confidence intervals for the identified QTL. (A,B) show QTL scans for bolting time on Chromosome 4; (A) Fixed effects (black) and mixed effects (red) logP, and (B) Hierarchical Bayes percentage of QTL variance (maroon); (C) fixed effects logP for the binary phenotype erecta on chromosome 2.

Our analysis correctly identified the genomic regions that contain the genes known to be responsible for the glabrous and erecta binary traits. For the erecta trait, the analysis identified a single QTL on chromosome 2 between 10.94 and 11.59 Mb (90% confidence region), EQ = 0.998, peak logP = 27.4 (Figure 8). The gene *ER* (*ERECTA*) is at 11.21 Mb and within 150 kb of the peak locus in the genome scan. Furthermore, analysis of those lines predicted to carry the Ler-0 haplotype at this locus (defined as the lines with P_i Ler_>0.8) identified all of the plants with the erecta phenotype. Likewise, analysis of the glabrous phenotype yields a single and narrow QTL on chromosome 3 between 9.94 and 10.79 Mb, which encompasses the gene *GL1* at 10.36 Mb. Analysis of the locus correctly shows that all variation is due to the haplotype from Wil-2, the only founder accession that is glabrous.

### Quantitative trait loci

Analysis of variation in bolting time in the greenhouse identified 4 QTL, on chromosomes 1, 4 and 5 (Table 3). Together they explain 63% of the total phenotypic variance in bolting time. The QTL on chromosome 4 (∼0.35 Mb) explains most of the variation (40%), and is likely to be caused by *FRIGIDA* (located at 0.26 Mb), a gene well known to affect flowering time [40]. The mean bolting times for each founder haplotype match the expected effect of *FRIGIDA*: haplotypes known to have a deletion that makes this locus non-functional [35] bolt earlier, and haplotypes known to have functional alleles flower later (Table S2). The QTL on chromosome 5 (∼3.5 Mb) is likely due to another gene well known to affect natural variation in flowering time: *FLOWERING LOCUS C* (located at 3.2 Mb). The QTL on chromosome 1 may be a complex of two linked QTL. The confidence interval for the first QTL on chromosome 1 (∼20.3 Mb) does not contain genes for which natural variation is known to affect bolting time. However, it is interesting to note that at 20.35 Mb is *ETHYLENE INSENSITIVE 5*, where T-DNA insertions have been previously observed to cause delay in flowering [41]. The second QTL on chromosome 1 (∼24.7 Mb) is likely due to *FLOWERING LOCUS T* (located at 24.3 Mb), a gene previously suggested to harbor natural variation that affects flowering time under long days [42]. Accordingly a co-localizing QTL was observed only under long day, but not short day conditions (see below). However, Interpretation of the QTL on chromosome 1 requires caution, since confidence intervals for linked QTL need not follow the same distribution as for an isolated QTL.

Flowering time was also phenotyped in growth chambers under long and short day conditions. Flowering time was measured as the number of days to flowering and the total number of leaves produced; leading to the identification of 2 QTL for each of the traits under long days, and 4 or 5 QTL under short days (Table 3). All QTL identified in the growth chamber were also on Chromosomes 1, 4 and 5. Some of these QTL co-locate with QTL identified in the greenhouse. However, a few locations suggest new candidate genes for natural variation in flowering time: On chromosome 4 (∼10.9 Mb), we found a QTL that explains a large proportion of the variation in flowering time and rosette leaf number under short day conditions only (16 and 21% of the variation respectively, Table 3). This QTL is in close proximity to *PHYTOCHOME E* (at 10 Mb); a locus where mutants that flower earlier under short day conditions have been previously observed [43]. The region on Chromosome 5 (∼0.76 Mb), which has QTL for flowering time in both long and short day, seems too distant to be still due to *FLOWERING LOCUS C*. A possible candidate gene for this region is *ETHYLENE INSENSITIVE 2* (located at 0.78 Mb), for which mutants with delayed flowering have been previously observed [44].

We also mapped QTL for vegetative growth rate (measured as the relative number of leaves, given their germination date), and the number of days between bolting and flowering. We found two QTL for each of these traits (Table 3), which together explain a small proportion of the genetic variance; approximately 28% in each case (Table 2). However, it is interesting to note that for both traits, a QTL located closely to *FRIGIDA* (on top of the chromosome 4) was found, which suggests that *FRIGIDA* may have a larger role in development timing, beyond just determining the onset of reproduction.

Finally, we detected two QTLs on chromosomes 3 and 4 for the number of days to germination. The QTL on chromosome 3 (∼15.9 Mb) is particularly interesting as it is located in the nitrilase gene cluster (NITRILASE 1, 2 and 3). These enzymes are thought to be involved in the production of the growth hormone indole-3 acetic acid, and NITRILASE 2 is specifically expressed in developing embryos. While the role of this gene in *A. thaliana* has been thought as being mainly in pathogen defense, nitrilase genes have been shown to be involved in seed germination in maize [45]. It is possible that this QTL collocates with a previously identified QTL, named DELAY OF GERMINATION 6 [46]. This QTL was identified as linked to the CAPS marker TOPP5, which is located at 17.2 Mb (the casual gene has not been identified).

### Online resources

All genotype and phenotype data and analysis software are available from our web site http://gscan.well.ox.ac.uk/arabidopsis. SNPs are also available from TAIR. The genome scans can be viewed using the browser http://gscan.well.ox.ac.uk/arabidopsis/wwwqtl.cgi which is an Arabidopsis-specific version of the genome scan browser GSCANDB [47]. The browser displays the genome scans and QTL, with genome annotations from TAIR, at arbitrary resolution.

## Discussion

We have described a new panel of genetically diverse and highly recombinant inbred lines of *A. thaliana*. Like other recombinant inbred lines they do not require repeated genotyping, and since unlimited replicates of each line can be grown, data for many traits can be accumulated, facilitating the study of trait correlations, genotype by environmental interactions, and the genetic basis of phenotypic plasticity. They represent a significant improvement over standard RILs descended from just two founders in that they capture more of the genetic and phenotypic variation present. Furthermore, they have a higher density of recombinants, which improves mapping resolution. We have shown how to take account of the increased genetic complexity in the analysis, and our results show that mapping accuracy and detection is much improved in the MLs when compared to traditional two-parent F2 and RIL mapping populations. Consequently, the MLs are an important new tool for the study of the genetic basis of plant growth and yield under multiple environments. Improved understanding of the genetic basis of such quantitative traits is important for the improvement of crop varieties, and to improve our basic knowledge of plant form, growth and development.

These lines are the first completed population of RILs descended from a large number of founders. Other populations, descended from eight founders are in production in *A. thaliana* [50], and *Mus musculus* (the Collaborative Cross [51],[52]). There are also ongoing efforts to produce similar populations in a number of crops including wheat, rice and sorghum with financial support from Generation Challenge Programme (http://www.generationcp.org, and Ian Mckay (NIAB), personal communication). The analysis of all these populations presents similar challenges, so lessons learnt with our lines should be valuable to the others.

Current strategies for QTL mapping in Arabidopsis range in complexity from F2 crosses, through panels of recombinant inbred lines and advanced intercross lines derived from two accessions [48], through combining multiple panels of RILs [27],[49], the MAGIC lines described here, and finally association mapping using a large collection of natural accessions. The MAGIC lines represent a compromise between the extreme simplicity of a diallelic system found in a RIL panel descended from just two progenitors with no population structure other than that due to segregation distortion [48], and the much greater complexity encountered in the natural accessions [13].

The power to detect a QTL in any mapping population depends on the phenotypic variance it explains, which ultimately depends on the frequency of the minor allele frequency at the QTL. The range in QTL minor allele frequency starts at 0.5 in diallelic populations, to at least 1/19 (0.052) in MAGIC (with mean value 0.22, if the genotyped SNPs are representative), to a potentially lower value in natural accessions (where many variants are unique to one accession [22]). Thus, to fine map QTL of small effect, a larger number of plants and genotypes are likely to be needed in a study using MAGIC lines or natural accessions, when compared to diallelic populations. Increasing replication within lines reduces non-genetic variance and improves power. However, even an infinite degree of replication cannot increase the fraction of variance explained by a single QTL to more than the fraction of total genetic variance it explains. Hence mapping QTL of very small effect and low minor allele frequency is likely to remain a challenge.

The genetic architecture of the traits we have mapped in this study range from simple – one QTL of large effect – to complex, with many QTL of smaller effect, some of which are physically linked. As expected, it is straightforward to map unlinked QTL, and the power and mapping resolution improves as the fraction of variance explained by the QTL increases. The dissection of multiple linked QTL is harder and the methodologies we have presented here could be improved. Nonetheless it is reassuring that the three methods we used – i.e., resample-based, hierarchical Bayesian and empirical Bayesian – all produce concordant QTL predictions. This suggests that the population structure of the MLs is not an impediment.

While previous RIL QTL studies have produced confidence intervals in the range of 2–20 Mb [53], the MAGIC lines generally produce much better resolution. The 90% confidence intervals were always smaller than 6 Mb, with some of the confidence intervals under 1 Mb; simulations indicate that for QTL with 10% effect size, the mean distance between the true QTL location and the midpoint of the marker interval containing the QTL peak is about 300 kb. Our results were in agreement with this expectation. For known QTL of large effect, as in the case of *ERECTA*, *GLABROUS*, *FRI*; the distance from the observed peak to the probable candidate genes was less than 300 kb. Certainly, in cases where these lines will be used for gene discovery, the size of the confidence intervals will still be an issue. However, we show that reasonable candidate genes are also found in close proximity to QTL even when a priori candidate genes were not known (e.g. in the case of *EIN 2*, *5* and *PHYE*).

We have shown that accuracy of about 300 kb is achievable in the ML using the statistical methodology described here. However, in association mapping the resolution is much greater (measured in the low tens of kb, or close to single gene) thanks to the very rapid decay in linkage disequilibrium with distance among wild accessions. Improvements in the power and mapping resolution of MLs are likely to come from using additional lines (currently in production) containing independent recombination events in which mapping resolution of under 200 kb should be achievable. We also expect to improve resolution by incorporating information about sequence differences between the founder strains (Resequencing the 19 founders of the MAGIC lines is now being conducted using sequencing by synthesis [54]). We plan to use merge analysis [55] to determine whether the allelic distribution of a variant across the 19 founders is consistent with the inferred phenotypic pattern of action, in order to test whether the variant could be causal for the QTL..

Finally, the combination of MAGIC and association mapping may prove fruitful. While association mapping may be able to identify QTL with better accuracy, the population structure observed among natural accessions requires much care to distinguish between true QTL and false positives [39]. In comparison, the structure of the MLs is relatively simple. If there are common variants in MLs and natural accessions, the MLs may provide an ideal material to verify QTL identified with association mapping.

## Materials and Methods

### Genotyping

We built a SNP database using information available at the time (2006/2007) from TAIR (http://www.arabidopsis.org), MSQT (http://msqt.weigelworld.org/), M. Nordborg's 1500 short sequences on 96 accessions [56] and http://walnut.usc.edu/2010); and unpublished data kindly provided by M. Koornneef (Max-Plank Institute, Cologne) and M. Purugganan (New York University, USA). From these data we selected 1536 SNPs for genotyping with the aim of covering the genome as uniformly as possible. SNPs that were predicted to be polymorphic between at least two accessions in our population and had a frequency of higher than 10% over all accessions previously genotyped were preferred. Since at the time of selection most genotypic information available was on accessions Col-0 and Ler-0, the selected SNPs are somewhat biased towards SNPs polymorphic for these accessions. The SNPs' flanking sequences were remapped to the Col-0 consensus sequences NC_003070, NC_003071, NC_003074, NC_003075, NC_003076 using BLAT [57] to obtain accurate localizations.

We genotyped 527 MLs and the 19 founders using the Illumina GoldenGate assay. SNPs with mean Illumina GenTrain quality score below 0.4 were removed and the few lines for which the overall genotype had GC quality score<0.4 were also removed. This left 1418 SNPs with an average missing data rate of 0.55%. We removed a further 115 SNPs that were found to be non-polymorphic among the founders and 43 SNPs with heterozyogosity exceeding 5%, leaving 1260 SNPs for analysis with mean spacing of 96 kb apart. For the QTL mapping all heterozygous genotypes were set to missing, resulting in a final missing data rate of 2.9%. We genotyped the founders in triplicate, and 53 MLs in duplicate; all 84074 repeated genotypes with QC scores>0.4 were concordant (the threshold of 0.4 was chosen to minimize discordant genotypes whilst maximizing the call rate). The complete list of SNPs is in Table S3, on our web site, and will be deposited with TAIR.

### Phenotyping

459 ML plus the 19 parental accessions were grown in five 2.5 inch pots filled with John Innes #3 compost in a greenhouse at the FIRS Botanical experimental grounds (Manchester), with 16 hours of artificial light/day, and the temperature set for 18°C. Each ML was planted into 5 pots, with each pot being randomly assigned to a tray. Trays were rotated throughout the greenhouse every week; and pots were reassigned to new trays approximately every 30 days. Due to space constraints in the greenhouse, phenotyping was performed in two separate batches. In each pot, we placed 3 seeds which were monitored daily for the day of cotyledon emergence (germination date). Two weeks later, only one of the seedlings that germinated was left at random. Plants were monitored daily and the date the flowering buds and open flowers were first noticed was recorded. Bolting time was calculated as the numbers of days between germination and the day the first flower bud was noticeable. We counted the number of leaves present in each plant 28 days after the seeds were sown to determine differences in growth rate. Growth rate for each plant was then calculated as the residual of the regression of number of leaves on germination date. At this time we also visually inspected the plants to determine if they were “glabrous”. After plants had flowered we scored them for their “erecta” phenotype.

Plants were also phenotyped in growth chambers at New York University using EGC walk-in chambers, under both long day (14 hrs light: 10 hrs dark) and short day conditions (10 hrs light: 14 hrs dark) at 20°C. Five individuals each for 360 MLs were grown in a randomized design in 72-cell growing flats, where each ML was randomly assigned to a given position in a flat (to avoid association between genotype and the microenviromental conditions experienced by a flat). The flats were repositioned within the chamber every 7 days and watered by sub-irrigation every 4 days. Flowering was determined as the number of days between planting and the primary inflorescence had extended more than 1 mm above the rosette, and by the number of rosette leaves present on a plant when transitioned to flowering. The possible effects of the tray was taken into account by using in the phenotype mapping the least square residuals, removing tray effects.

### Heritability analysis

To determine the fraction of phenotypic variation due to genetic variation, we estimated the heritability among MLs by fitting the random effects model:
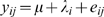
where 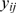 is the phenotype measured on the *j*th replicate plant of line *i*, 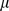 is the overall mean, 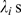 the phenotypic effect of the genotype of each line *i*, which is modeled as a random variable drawn from a normal distribution with mean 0 and variance 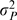, and 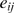 is the variation due to non-genetic causes, which is assumed to be normally distributed with mean 0 and variance 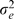.

We report two heritabilities: (i) the heritability of individual plants
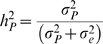
and (ii) the heritability of the phenotype averaged across replicates within MLs:
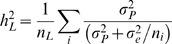
where *n_i_* is the number of lines and *n_i_* the number of replicates within line *i*. Both are computed by substituting the maximum likelihood estimates 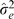 and 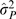.

### QTL mapping

A hidden Markov model (HMM) is used to make a multipoint probabilistic reconstruction of the genome of each ML as a mosaic of the founder haplotypes [37]. The ML breeding design means that each genome is made up of segments of the founder genomes, with a transition between founders occurring whenever a recombination has occurred. Diallelic SNPs cannot distinguish between all founders so information from neighboring SNPs is used to compute the posterior probability 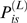 that at a given locus *L*, the ML *i* is descended from founder *s*. Here, a locus is defined to be the interval between two adjacent genotyped SNPs, labeled by the name of the left-hand SNP.

The HMM makes the following approximations and assumptions (i) the genome of each ML is completely homozygous (we ensure this by deleting the small fraction of heterozyous genotypes). (ii) The effective number of generations, 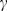, since the cross was originated is 6, comprising 4 generations of crossing during the funnel breeding phase, plus two effective generations from the selfing phase (because on average only two informative meioses per Morgan are accumulated during selfing). (iii) The identity of the founder in a given segment in the mosaic is uncorrelated with other segments for that individual (iv) the length of segment in centiMorgans is exponentially distributed with mean length 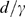, where 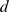 is the genetic length of the segment, corresponding to a Haldane mapping function with 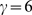 fold map expansion.

Evidence for a QTL within each locus is first evaluated when the effects of all other QTL are ignored; this step corresponds to a standard genome scan. Suppose there is a QTL segregating at locus *L* in which the phenotypic effect due to founder haplotype *s* is 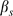, the phenotype 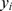 in ML *i* is modeled as
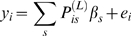
where 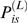 is the HMM probability computed in step 1. This may be rewritten as
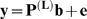
(1)where 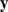 is the vector of phenotypes, 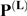 is the matrix representing 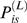 and 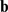 the vector representing 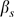. The hypothesis that there is no QTL is equivalent to testing if the 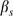 are identical, by fitting a fixed-effects linear model with up to 18 degrees of freedom and performing an ANOVA. The statistical significance of the genome scan at each locus *L* is summarized by logP = −log_10_ (ANOVA P-value), so that logP increases with the significance of the QTL.

This method is most powerful when the probabilities 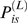 are either 0 or 1, but it extracts useful information even when this is not the case provided they are not all equal. Many phenotypes that are not normally distributed (e.g. binary and survival traits) can be accommodated by extending the formalism to a generalized linear model framework (see [38] for details).

The evidence for each QTL is re-evaluated in the context of other segregating QTLs, by averaging over many likely multiple QTL models. To do this, a random subsample of 80% of the total number of MLs is made, and a multiple QTL model created by forward selection, adding loci to the model until it is not possible to improve the fit of the model significantly. The locations of the QTL are recorded and the process is repeated 500 times. Each re-sampling of the data produces a different multiple QTL model, and the fraction of models containing a given QTL is the measure of support for the QTL (the Resample-based Model Inclusion Probability, or RMIP). Clusters of nearby loci with positive RMIP are treated as the same QTL; a dynamic-programming algorithm is used to identify the clusters, and the value reported for the QTL is the sum of the constituent RMIP values, the expected number of QTL within the region, which we call the Expected QTL (EQ). If the EQ>1 then some multiple QTL models contain more than one QTL for the same region, suggesting QTL is likely to contain several linked loci. The multiple QTL mapping was performed using the R program bagphenotype (http://www.well.ox.ac.uk/~valdar/software/bagphenotype).

### Estimation of founder strain effects at a QTL

Least-squares estimates 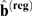 from fitting the fixed-effects multiple regression Eqn 1 at a QTL are unbiased but numerically unstable whenever some founders are almost indistinguishable at a locus. This results in near multicollineanity in the matrix 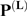 and in pairs of estimates of very large magnitudes but opposing signs, which cancel each other out, and therefore are hard to interpret biologically.

Instead, stable unbiased estimates are obtained by multiple imputation [58]; 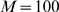 design matrices 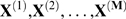 are sampled from the distribution 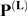 such that each matrix 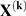 has the same dimension as 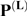, precisely one element in the *i*'th row is 1 and the rest are 0, with 
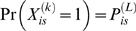
. Next, the linear model 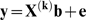 (in fact a one-way analysis of variance) is fitted to each imputed matrix, giving a sequence of least squares estimates 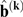, each with an error following a t-distribution (with degrees of freedom that can vary between imputations depending on the rank of 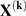). The distribution of the imputed strain effects 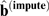
is estimated as the average of the distributions of the 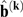 e.g.
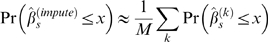
Binary-valued phenotypes 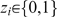 are treated in a conceptually similar way, with 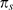 modeling the penetrance when the founder strain is *s, i.e* 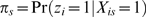. Then 
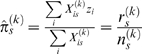
, say, and 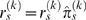 is distributed as 
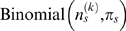
. Then
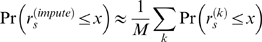
and the imputed penetrance is defined as
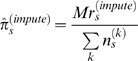



### Power simulations

To understand the locus-specific properties of QTL mapping using the MLs we estimated the power to detect and fine map a single QTL of varying location and effect size. In each simulation, the locus position *L* was selected randomly and a diallelic QTL simulated in which 4 randomly chosen accessions carried the minor allele and the remainder the major allele (corresponding to a minor allele frequency close to the observed average value of 0.22). The unobserved founder strain genotypes at the QTL were simulated by sampling from the HMM distribution 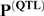 for the marker interval containing the QTL. The phenotypic effect due to each allele was adjusted so that the QTL effect size (the fraction of variance explained by the QTL in the mapping population) equaled a target value in the range 5% to 30%. Then a genome scan was performed and the location of the most significant locus with the maximum logP recorded. If this maximum was within 3 Mb of the QTL then the simulation was classified as a success and the mapping resolution computed as the displacement between the true QTL location from the midpoint of the marker interval containing the maximum logP. We simulated over 350,000 QTL. We then divided the genome into 1 Mb segments and, within each segment and for each QTL effect size, estimated power as the fraction of successful simulations for QTL in the segment, and mapping resolution by the distribution of QTL displacements for the successful simulations. For each simulation we also recorded the maximum logP.

We used those 285399 simulations in which the QTL lay outside of the centromeres, was detected at genome-wide significant (logP_MAX_>3) and mapped to within 3 Mb of the true location, to estimate the distribution of mapping resolution as a function of logP_MAX_. If x_QTL_ is the true location of the QTL and x_MAX_, logP_MAX_ are the location and logP -value of the maximum in the genome scan, then we estimated the empirical cumulative distribution function Pr( | x_QTL_−x_MAX_|<*d* | logP_MAX_) from those simulations whose global maximum logP_SIMULATED_ was close to logP_MAX_, specifically for which |logP_MAX_ − logP_SIMULATED_|<0.25. No attempt was made to localize a QTL within a marker interval, because the mapping resolution is generally poorer than the average spacing between markers.

The null distribution of the genome-wide maximum logP was estimated from 10000 simulations when no QTL was present. To understand the impact of large effect QTL on inflating background values of logP, we compared, for the set of simulations with a single QTL explaining 15%of the variance, the distribution of the maximum logP on chromosomes excluding that containing the QTL with the null distribution.

To estimate power and mapping resolution in the complete MAGIC cohort of 1026 lines, we used the program valbreed (http://www.well.ox.ac.uk/~valdar/software/valbreed) to simulate the genomes of the complete population from the 19 founders, using the observed genotypes in the founders. We then simulated 10,000 5% and 10,000 10% QTLs, and estimated power and mapping resolution in this simulated population in the same way as for the real lines.

### Comparison with Empirical Bayes and Hierarchical Bayes QTL mapping methods

We implemented an Empirical Bayes mixed effects QTL mapping method that takes into account the MAGIC population structure [13],[24]. The fixed effects model (Eqn 1) is augmented by an additional random effect representing the increased phenotypic similarity expected between lines descended from the same F4 cross. Thus, if 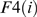 is the identity of the F4 cross for ML *i*, then
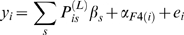
(2)where 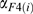 is distributed as 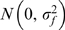.. The variance 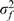 summarizes the effects of other QTL, so
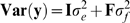
where the matrix 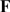 is defined as
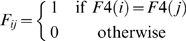



This is both a linear mixed model and an Empirical Bayes model, fitted using the lme4 R package. Statistical significance of the test for presence of the QTL is assessed as the logP of the likelihood ratio test statistic comparing the fit of the model to the null model where 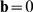. Genome-wide significance thresholds are estimated by simulating phenotypes from the null model and performing 200 genome scans. Full details of the method are described in Valdar et al [37].

We also developed and implemented a Hierarchical Bayes method that treats 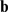 as a random effect (C Durrant; manuscript in preparation). The rationale for this approach is that a large number (up to 18) of degrees of freedom are required to fit a fixed-effects model, but we would expect that in many QTL the causative DNA polymorphism will have far fewer alleles. In our hierarchical Bayesian re-interpretation of Eqn 1, the distribution of the founder effect 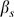 is modeled as 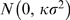, where 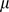 is the overall mean, 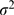 is the total phenotypic variance and 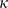 measures the fraction of the variance explained by the QTL. The prior distribution of 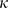 is Uniform[0,1], a non-informative prior for a proportion. The resulting estimate of the proportion of variance due to the locus estimates the true variance between the founder strain effects, independent of the observed sample frequencies of the founder strains. Hence this estimate will not always match the one-way ANOVA, which is dependent on the sample frequencies.

If the HMM probabilities are all either 0 or 1, then these priors produce a joint posterior distribution which factorizes completely and avoids the need to use MCMC techniques. If we consider the HMM probabilities as the posterior distribution for the founder strains for each individual at that locus, we can extend the factorization of the joint posterior to include the case when the HMM probabilities are not all 0 or 1. This results in the parameter estimates being averaged over all possible combinations of founder strains at that locus, a Bayesian analogue of the multiple imputation approach described above. The mode of the posterior distribution of 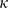 is used as the point estimate reported for the genome scan, rescaled to represent the percentage of the phenotypic variance due to the locus.

## Supporting Information

Table S1 List Of MAGIC lines, the F4 family they are derived from, and the accessions that entered each pedigree.
(0.08 MB XLS)

Table S2 Phenotypic variation among accessions in bolting time, known *FRIGIDA* and *FLOWERING LOCUS C* allele type, and haplotype effects at bolting QTL.
(0.02 MB XLS)

Table S3 List of all SNPs scored in the 19 founder accessions and MLs. All genotypes are homozygous and indicate by a single letter.
(1.40 MB TXT)
